# A Strain-Based Method to Detect Tires’ Loss of Grip and Estimate Lateral Friction Coefficient from Experimental Data by Fuzzy Logic for Intelligent Tire Development

**DOI:** 10.3390/s18020490

**Published:** 2018-02-06

**Authors:** Jorge Yunta, Daniel Garcia-Pozuelo, Vicente Diaz, Oluremi Olatunbosun

**Affiliations:** 1 Department of Mechanical Engineering, Research Institute of Vehicle Safety (ISVA), Universidad Carlos III de Madrid, Avd. De la Universidad, 28911 Leganés (Madrid), Spain; dgramos@ing.uc3m.es (D.G.-P.); vdiaz@ing.uc3m.es (V.D.); 2 School of Mechanical Engineering, University of Birmingham, B15 2TT Edgbaston, UK; o.a.olatunbosun@bham.ac.uk

**Keywords:** intelligent tires, strain gauges, fuzzy logic, tire slip, lateral friction coefficient

## Abstract

Tires are a key sub-system of vehicles that have a big responsibility for comfort, fuel consumption and traffic safety. However, current tires are just passive rubber elements which do not contribute actively to improve the driving experience or vehicle safety. The lack of information from the tire during driving gives cause for developing an intelligent tire. Therefore, the aim of the intelligent tire is to monitor tire working conditions in real-time, providing useful information to other systems and becoming an active system. In this paper, tire tread deformation is measured to provide a strong experimental base with different experiments and test results by means of a tire fitted with sensors. Tests under different working conditions such as vertical load or slip angle have been carried out with an indoor tire test rig. The experimental data analysis shows the strong relation that exists between lateral force and the maximum tensile and compressive strain peaks when the tire is not working at the limit of grip. In the last section, an estimation system from experimental data has been developed and implemented in Simulink to show the potential of strain sensors for developing intelligent tire systems, obtaining as major results a signal to detect tire’s loss of grip and estimations of the lateral friction coefficient.

## 1. Introduction

As the only part that keeps the contact between the vehicle chassis and the road, tires are a key factor for electronic control systems as well as for comfort and fuel consumption, among others. Despite the fact that current tires perform well in a huge variety of situations, they are just passive elements that do not contribute actively to driver or vehicle control systems to improve the driving experience and traffic safety. All these reasons encourage researches and companies to invest time and efforts in the development of an intelligent tire that works as a source of information for drivers and active control systems such as the Traction Control System (TCS) or the Electronic Stability Program (ESP) to intervene before a crash occurs.

Up to now, the Tire Pressure Monitoring System (TPMS), which was introduced in the market in 2002 [1] and provides information about tire inflation pressure, is the only system installed in tires that gives the drivers some kind of information about tire working conditions while driving. Nevertheless, real-time monitoring of other parameters like forces at the tire-road interface, wear and tire-road friction, which also depend on uncontrollable environmental factors, is the main obstacle as well as the ultimate goal of the intelligent tire development. 

**Figure 1:**
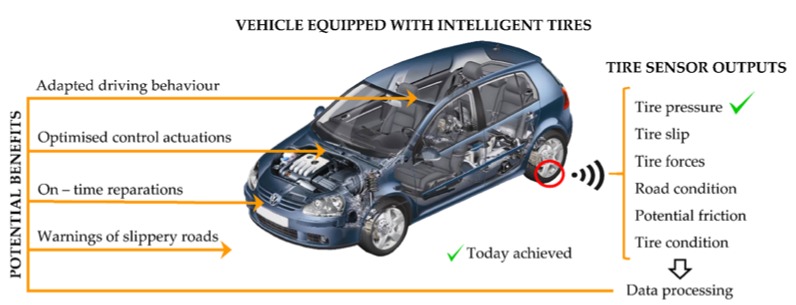
Intelligent tire’s potential benefits [2,3].

During the past 20 years, many researches have been made on the field of the intelligent tire [2], concluding that, although the TPMS was a good advance, the scope of the intelligent tire is much more ambitious than TPMS, as shown Figure 1.

As is well known, driver actions in emergency situations can subject tires to forces greater than they can handle. As a consequence, the tire saturates and is unable to transfer enough lateral or longitudinal tractive effort, so the vehicle becomes unstable and probably uncontrollable. Due to nonlinear tire-road contact features, this situation may result in a traffic accident, more so if unsuitable road conditions are encountered like wet or muddy surfaces where friction is changing [4]. Therefore, the possibility of tires playing an active role in in mitigating this kind of situation is subject to the potential ability of intelligent tires to monitor some essential parameters about their working conditions. 

**Figure 2:**
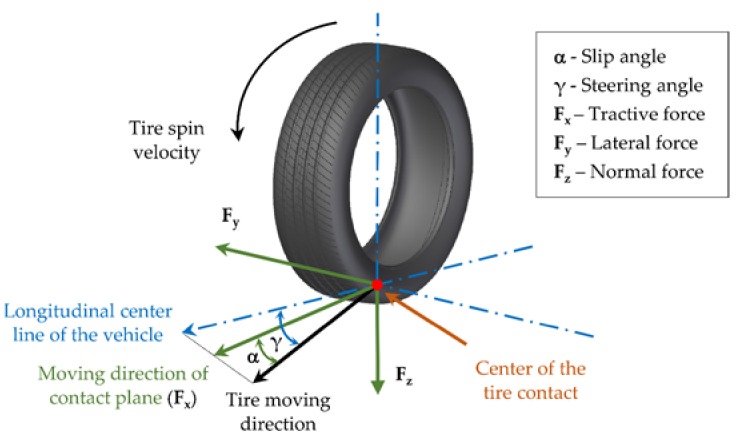
Tire slip angle and steering angle.

Regarding parameters that are restricted by the driver’s action, there are two important angles to take especially into account: the wheel steer angle (γ), which is usually explained as the angle between the tire moving direction and the longitudinal center line of the vehicle, and the slip angle (α), which is the angle between a rolling wheel’s actual direction of travel and the direction towards which it is pointing [5]. These angles and the different forces generated in the contact patch area are represented in Figure 2. In this paper, the tire slip angle will be used in the last section as a function of the wheel steer angle as a tool to detect that the tire is about to lose adhesion.

Several researches have concluded that the estimation of the slip angle is a vital point to improve the behavior of the tire and the vehicle [6,7]. It was found that the slip angle is directly related to the lateral force, which is usually defined as the force that the tire transmits to the road in the lateral direction, generating the required grip [8,9]. For this reason, a large and growing body of literature has also investigated their estimation [10,11,12,13]. Overall, these researches indicate that lateral and longitudinal tire-road forces depend on different parameters regarding the working conditions or the tire properties. For this reason, the relation between them and the lateral force is quite complicated and nonlinear. In cornering conditions, a car’s tires deform laterally and longitudinally in the contact patch area [14]. Thus, tires must generate lateral forces to drive the car along a certain path. If the tire is capable of transferring these forces to the road, the tire does not slide and, as a result, the car follows the desired path.

On the other hand, forces generated in the tire-road interface are part of the parameters whose values cannot be controlled solely by the driver’s action, because they depend on other uncontrollable factors, for instance, temperature or road conditions. The relation between those forces, which are represented in Figure 2, and slip angle, determines the vehicle’s dynamics. Regarding lateral dynamics, the typical tire lateral characteristic curve as a function of the slip angle is usually divided into three regions: linear/elastic, transitional, and frictional [14]. In the linear section, the tire tread is not sliding on the road at any point in its contact. However, at higher slip angle some parts of the contact patch area are sliding, generating lower lateral force. For better understanding of this work, it is important to clarify that the term “sliding” does not imply that the driver realizes that the tire is not working at the limit of adhesion or the vehicle is not following the desired path, since during the driving experience the influence of the loss of adhesion is not easily observable when it has just started. For this reason, to detect that the lateral force is decreasing could be a key information for the driver and the active safety control system before the situation becomes more dangerous.

In addition, lateral force depends mainly on vertical load as well as slip angle [14]. Although these curves are specific and different for each tire, it is generally observed that lateral force increases as vertical load increases, and therefore, the required adhesion. Besides, lateral force stabilizes when it reaches its maximum at a certain slip angle. In this work, some lateral force vs. slip angle curves have been experimentally obtained in order to get a mathematical expression that allows the lateral force to be calculated as a function of the vertical load and the slip angle.

In the same manner as for vertical load, lateral force and slip angle, the influence of other mechanical tire features such as inflation pressure or rolling speed on tire strain behavior, which can make possible the estimation of tire behavior and provide information for vehicle active control systems and drivers, is also analyzed in this paper. Most of these tire features have been analyzed in several works related to the intelligent tire. Overall, they analyze the tire’s deformation by measuring sidewall deflection, tire tread deformation, acceleration, etc. by means of different sensors such as Surface Acoustic Wave (SAW) sensors [15,16,17], optical sensors [18], piezoelectric sensors [13,19], accelerometers [20,21] or capacitive sensors [22,23,24,25]. Although all of them demonstrated the capability to provide information about tire working conditions from tire behavior, they usually have some limitations such as energy consumption, robustness or dependency of the tire-rim relative position.

There is a large volume of published studies indicating that strain sensors meet the requirements to achieve an advanced intelligent tire system from strain measurement [26,27,28]. For instance, Morinaga et al. [9] installed strain sensors on the inner liner of the tire tread, obtaining significant, stable and precise information about tire forces generated in the tire-road contact. In addition, strain sensors are less expensive than other sensors to measure tire deformation, which is associated with most of the tire working conditions. The price of strain gauges as well as the robustness thereof have demonstrated that they are suitable for developing the intelligent tire. 

In addition to the discussion about what sensor technology is more suitable for developing the intelligent tire, it should not be forgotten that its future viability is subject to the real-time application. To do this, many obstacles must be surmounted, such as the compatibility of the sensors with tire rubber characteristics (for example, stiffness issues), data transmission [29] or economic issues relating to the cost of the sensors, but the main obstacle has always been to meet the power requirements of all the electronic components. This aspect has been explored recently in some researches [30]. Yilmazoglu et al. [31] proposed an optimized material system that could reduce the power for sensors implementation. Moreover, Jousimaa et al. [32] recently used a piezoelectric energy harvesting system, storing energy to supply energy for low-power electronic devices. 

Finally, in addition to the multi-disciplinary knowledge about sensor technology, data analysis, tire characteristics, etc. that is required for the intelligent tire development, the involvement of computational methods is absolutely necessary to take advantage of the sensor’s measurements. Overall, most researches focus on estimating the tire-road friction coefficient, first, by characterizing the road surface from tire behavior or using tire or vehicle models, and then implementing them in estimation tools such as Kalman filters [33,34,35,36,37].

In this paper, a series of experiments by means of strain sensor equipment and an indoor tire test rig have been carried out. These devices were selected to measure strain dynamic behavior based on tire working conditions, which could help towards developing intelligent tire systems in the near future. Thus, this paper is devoted to provide a strong experimental base with test results under different tire working conditions. This work is a continuation of the experiments carried out to measure the tire’s deformation at the inner surface of the tire tread and estimate tire working conditions by means of fuzzy logic [26,28,38]. It concentrates on the influence of pressure, rolling speed, vertical load and slip angle on maximum strain values and lateral force measurements to elucidate how maximum strain data and lateral force are interconnected. In the last section, an experimental model of the lateral force behavior and previous estimations of slip angle and vertical load [38] are used to detect the tire’s loss of grip and calculate the lateral friction coefficient, showing the proof of concept of a proposed Slip Detection System (SDS) as well as the potential of strain sensors for the development of intelligent tires.

## 2. Materials and Methods 

### 2.1. Strain-Based Experiment Setup

**Figure 3:**
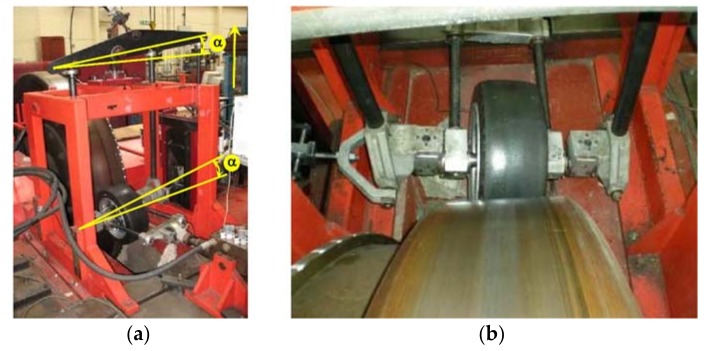
(**a**) Indoor tire test rig setup; (**b**) Tire working under cornering conditions.

An indoor tire test rig which allows the speed, vertical load and slip angle to be varied has been used for the experiments. The test system setup (including the indoor tire test rig equipped with the tire prototype) is shown in Figure 3a. The drum’s curved surface has an insignificant effect on the results because of its large diameter (2.44 m) which ensures that there is only slight difference between a flat road and the large drum for tire/road contact. The error in contact length is less than 0.1% [39]. The test rig allows the lateral force to be monitored when the slip angle is changed, as shown in Figure 3b.

**Figure 4:**
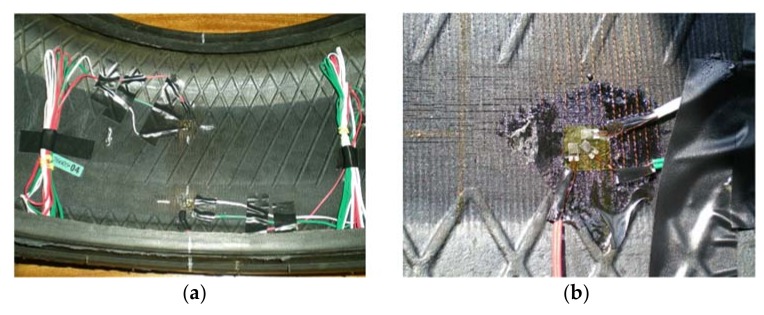
(**a**) Strain gauges’ setup; (**b**) Multiaxial gage example.

Some strain-based intelligent tire systems that use strain sensors have been suggested in the past [9,26,28]. They were attached on the inner liner of the tire tread, demonstrating that a strain sensor installed near the contact patch provides more detailed and prompt information about local frictional condition between the tire and the road. In this study, some strain gauges are mounted on the inner liner surface of the tire tread in longitudinal and lateral directions, as shown in Figure 4a. 

A data acquisition system with high resolution is especially necessary because of the possibility that the tread thickness attenuates the strain measurements. Figure 4b shows the installation of the rectangular rosette strain sensors. The manufacturer recommended adhesive for rubber materials was used, therefore, local stiffening effect caused by the strain gage can be neglected. The strain gage’s length is 2 mm with gage resistance 120 Ω. The resolution of the strain measurement is 0.001 µε.

**Figure 5:**
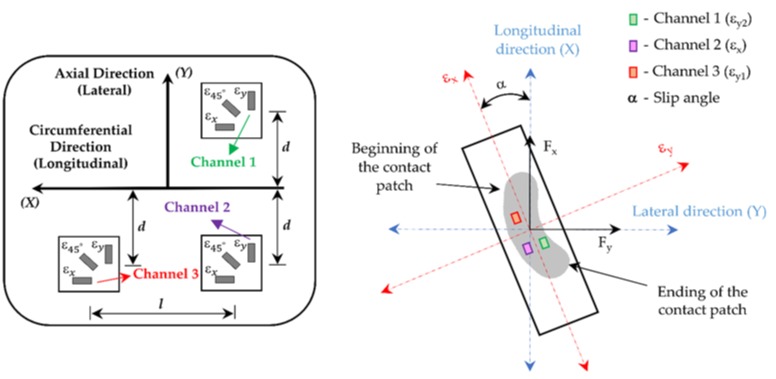
Strain gauges’ location scheme.

These strain gauges, which are widely used for large strain measurements, were attached at different points of the inner liner of the tire tread in order to measure lateral (ε_y2_—Channel 1 and ε_y1_—Channel 3) and longitudinal (ε_x_—Channel 2) strain, as shown in Figure 5. Regarding the position of the strain gauges, note that there are three multiaxial strain gauges located symmetrically to tire central line, two of them in the same cross section and the third one separated by 123.75° of angular rotation. The distances “d” and “l” are about 0.040 m and 0.515 m, respectively.

**Figure 6:**
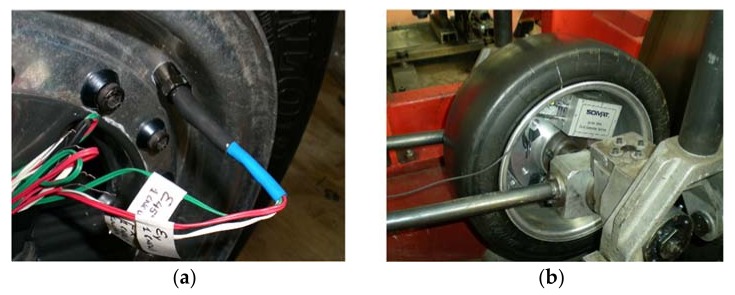
(**a**) Installed valves on the rim; (**b**) Installed SoMat^®^ 2000 in the wheel.

In order to pack and route the lead wires from the strain sensors to the outside of the rim, three additional valves were installed on the rim, as shown in Figure 6a. The lead wires were routed through the valves and all the holes and joints were sealed with adhesive to avoid any possible air leaks, leaving the system for 72 h to check the seal reliability. These valves allow the connection between strain sensors and data acquisition system. Although several channels are illustrated in Figure 5, not all of them were analysed in detail due to two principal reasons: some wires were damaged when passing them through the valves and others showed high similarity in the measurements and do not seem to provide interesting additional information.

The SoMat^®^ 2000 Field Computer (Somat Corporation, Urbana, IL, USA), which is suitable for portable data collection, was used as a data acquisition system. The hardware of the system consists of a Processor module, which has the microprocessor data acquisition system, and a Power/Communication module, equipped with batteries, as shown in Figure 6b.

SoMat^®^ 2000 has one Wheatstone strain bridge for each strain sensor, which can be used in a quarter, half or full bridge configuration. The device was driven by TestPoint® software (Capital Equipment Corporation, Norton, MA, USA) for Windows (WinTCS). When a test starts, the data acquisition module (i.e., the strain gauges module) is connected to the strain sensors. Secondly, the user can download the test specification, designed using WinTCS, from a computer to the SoMat^®^ 2000 to initialize and run it. By sampling the electrical analogue signals, the data acquisition system can store and manipulate digital data as bytes of binary digits. Although in this work the sampling frequency was set to be 1000 Hz, the SoMat^®^ 2000 Field Computer can provide a sampling frequency from 0.0005 to 5000 Hz, which ensures that the test strain data resolution is adequate to monitor enough strain points per tire revolution. 

However, it should not be forgotten that the data acquisition can be influenced by some factors which are usually divided into two groups: properties of the data acquisition system and the tire properties (such as tire radius or speed). Regarding properties of the data acquisition system, a large number of data channels would increase the amount of data acquired, however, more memory would be needed and the available test time would decrease. The maximum speed used in this work was 50 km/h, which is suitable for the sampling frequency used. The working range of the SoMat^®^ 2000 strain gage module used covered from −5000 to 5000 µε.

The test carried out consisted of studying the tread’s dynamic behavior by changing test conditions in order to determine the influence of pressure, vertical load, speed and slip angle on tire strain. To do this, measurements from strain sensors were downloaded to a computer after the tests and analyzed. A DUNLOP SP SPORT 175/505 R13 (tubeless) slick radial tire was used in the tests. This type of tire, which usually works under low vertical loads and inflation pressures, is used for the Formula SAE vehicles.

### 2.2. Test Conditions

The operational range of parameters used for the cornering conditions were:

- Tire inflation pressure: 0.8–1.4 bar;
- Tire preload: 250–1000 N;
- Tire speed: 10–50 km/h;
- Tire slip angle: 0–14°.

It should be underlined that trials between 0° and 10° have been performed every 2 degrees, but it was decided not to include data at 12° as the potentiometer, which supplies the system, did not provide a stable signal for that slip angle. Instead, it was decided to conduct trials at 13° as exception. Finally, the effect of temperature or inflation pressure changes in the tire between non-rotating and rotating phases are not considered, being the recommendations of the strain gauges’ manufacturer 23 °C and 50% relative humidity.

## 3. Results

### 3.1. Experimental Data Analysis

The influence of lateral force and the tire working conditions considered during the experiments on strain data is studied in this work by the detailed analysis of experimental data. 

First of all, it should be mentioned that although some of those working conditions, mainly tire parameters, could be measured using several sensors, the ultimate goal of the proposed tire prototype is to provide a platform for reliable and accurate measurement of more tire operating parameters/variables using the minimum number of sensors possible.

The variation in the obtained strain data may also be useful information to identify the variation of tire working conditions. Since the tire’s deformation occurs due to the requirements of the working conditions, the variation and the characteristics of the obtained strain data related to the operating conditions play a significant role in the estimation of the tire’s dynamic behavior, tire tread wear and other characteristics [40].

Before starting the experimental data analysis, representative data curves were calculated considering the average strain for each point of the tire perimeter by averaging the separate cycles, which were obtained under the same driving conditions. Then, lateral force, slip angle and tire surface deformation can be contrasted. 

**Figure 7:**
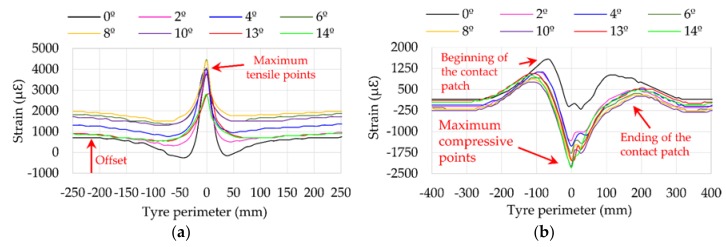
(**a**) Strain data curves in channel 2 (ε_x_) at 1 bar, 750 N, 30 km/h; (**b**) Strain data curves in channel 3 (ε_y1_) at 1 bar, 750 N, 30 km/h.

Strain curves have some characteristics that must be mentioned. As indicated above, data from three different channels were obtained, one in the longitudinal direction (ε_x_) and two in the lateral direction (ε_y1_ and ε_y2_), as shown in Figure 5. Note that the channels 2 and 3 are located on the outer part of the contact patch while the channel 1 is on the inner part of the contact patch (see Figure 5). Nevertheless, past researches [38] showed that the most significant information is measured by channels 2 and 3. Figure 7a,b show examples of strain curves measured by these channels for different slip angles. 

It was also revealed in [28] that in cornering conditions the representative points are the maximum tensile points in channel 2, which correspond to the center of the contact patch, as well as the maximum tensile values at the beginning and ending of the contact patch in channel 3. In addition, Figure 7a shows that the offset value of the signal is different depending on the conditions, which is also interesting as a source of information when the sensor is far from the contact patch. 

Finally, although it seems that the maximum compressive points (see Figure 7b) could also be useful to extract information about tire working conditions, previous studies reveal that these maximum points are not linear for all test conditions and fluctuations exist that hinder the extraction of useful information, thus, it is not considered to be as useful as the others are.

In the following sections, the influence of rolling speed, inflation pressure and vertical load related to slip angle and lateral force is shown, considering the maximum strain peaks and the offset values of the strain signal.

### 3.2. Influence of Speed

**Figure 8:**
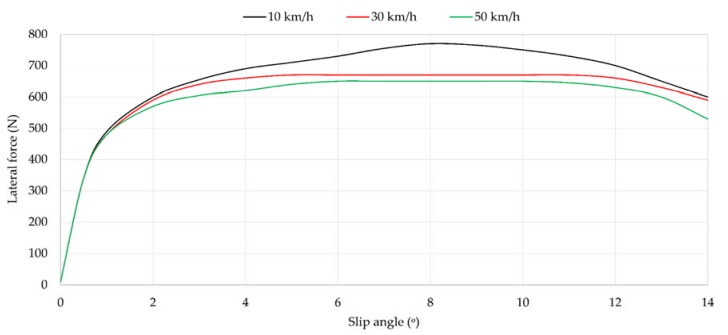
Influence of speed at 0.8 bar, 750 N. N: Newtons.

Figure 8 shows the variation of lateral force as slip angle increases at 10, 30 and 50 km/h. As expected, it can be clearly split into three parts. From 0° to 3°, which correspond to the elastic region (see Figure 8), the curves are practically overlapped. The second part covers from 3° to 9°. The maximum lateral force is reached at 8°, approximately. 

**Figure 9:**
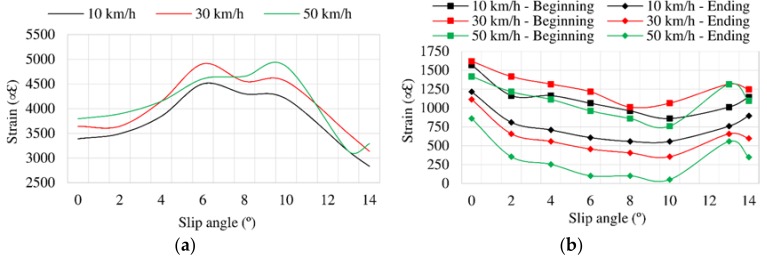
(**a**) Maximum strain peaks in channel 2 (ε_x_) at 0.8 bar, 750 N; (**b**) Maximum strain peaks in channel 3 (ε_y1_) at 0.8 bar, 750 N.

The three characteristic regions can be easily identified in Figure 9a,b, which represent the variation of maximum tensile strain data as slip angle increases. Strain data decrease drastically from 10° approximately for any speed. In the same way, data trend also changes in Figure 9b from 10° but in this case strain data increase. This fact reveals that the Poisson effect appears clearly in the tread tire, since when maximum strain values decrease in longitudinal direction from 10° (channel 2), they increase in lateral direction (channel 3). Note that this phenomenon occurs both at the beginning and ending of the contact patch. 

It is also interesting to underline from Figure 9a that between 6° and 10° maximum strain data values are reached. Since these points belong to the transitional range (i.e., the optimal working range), it can be assumed that when maximum strain values are higher, the maximum required grip is obtained. However, in contrast to Figure 9, the variation of maximum tensile strain is not monotonic, especially in the longitudinal direction where, for instance, at 10 and 30 km/h the maximum tensile peaks occur at 6° while at 50 km/h it is found at 10°. It may be due to the behaviour of the tire, a non-linear system, which is influenced by the rubber tribology behaviour, the study of which is not an objective of this paper. Moreover, there appears to be some relation between maximum strain values and maximum lateral force, since from 10° the lateral force reduces and maximum strain values change notably. 

### 3.3. Influence of Inflation Pressure

**Figure 10:**
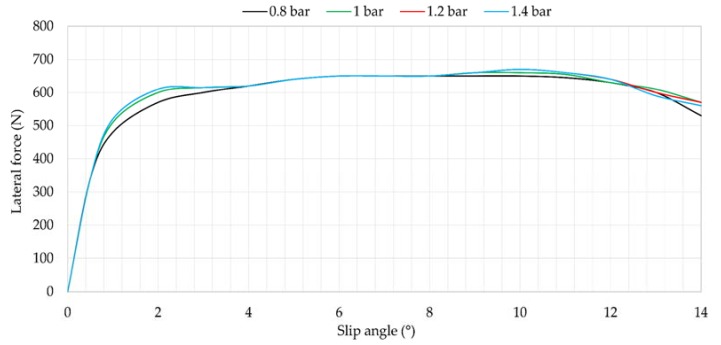
Influence of inflation pressure at 750 N, 50 km/h.

Figure 10 shows the influence of the slip angle on the lateral force at 0.8, 1, 1.2 and 1.4 bar of inflation pressure. Note that, curves from 4° to 8° are practically overlapped, so inflation pressure does not have too much effect in this region. However, the influence of tire pressure could be important from 10°, when the tire is not working at the limit of adhesion.

**Figure 11:**
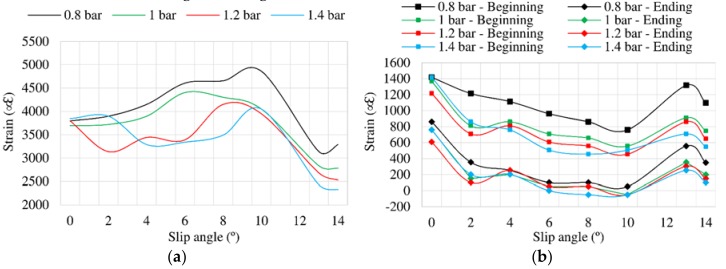
(**a**) Maximum strain peaks in channel 2 (ε_x_) at 750 N, 50 km/h; (**b**) Maximum strain peaks in channel 3 (ε_y1_) at 750 N, 50 km/h.

Maximum strain values are represented in Figure 11 for channels 2 and 3. Although there are fluctuations to 10°, from that point the data trend changes notably. These points correspond to the points at which the tire begins sliding.

**Figure 12:**
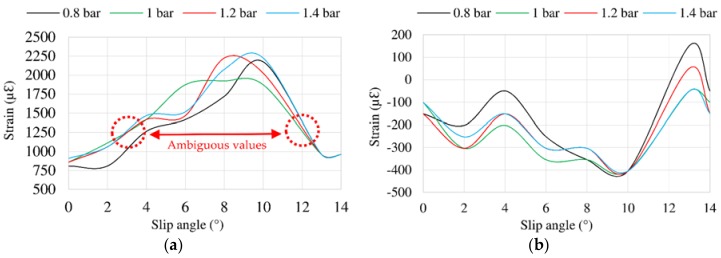
(**a**) Offset values in channel 2 (ε_x_) at 750 N, 50 km/h; (**b**) Offset values in channel 3 (ε_y1_) at 750 N, 50 km/h.

Figure 12 represents the offset variation for inflation pressure changes. In this case, there seems to be a relation between offset values in longitudinal direction (see Figure 12a) and offset values in lateral direction (see Figure 12b) and the maximum lateral force, that is reached around 9° in Figure 10, just before the tire starts sliding. In addition, the relationship between Figure 12a,b can be explained by the Poisson effect, especially when the tire is sliding.

Finally, it is observed in Figure 12a that some strain data are the same for different slip angles, for instance, for 3° and 12° (i.e., ambiguous values). This fact will play a key role for the fuzzy logic implementation in the last section of this paper.

### 3.4. Influence of Vertical Load

**Figure 13:**
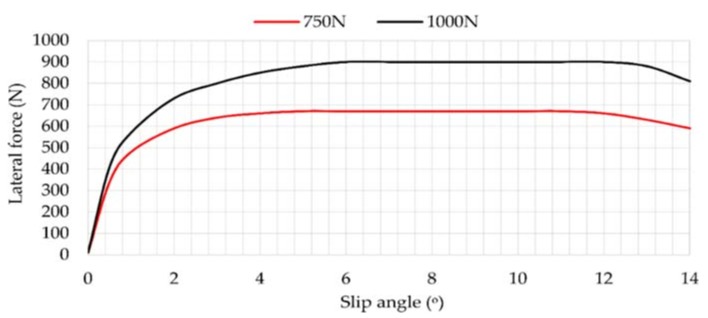
Lateral force vs. slip angle at 0.8 bar, 30 km/h.

Figure 13 illustrates the influence of the slip angle on lateral force for vertical loads of 750 and 1000 N. It should be mentioned that, although tests at 250, 500, 750 and 1000 N were carried out for obtaining results from strain sensors, the vertical load actuator that applied a specific value of vertical load was not capable of keeping a constant value when it was not very high, so lateral force values were not as accurate as in case of 750 and 1000 N. For this reason, lateral force results at 250 and 500 N have been neglected in this case.

Different from Figure 8 and Figure 10, the curves are much more stable and are well separated in Figure 13. In addition, they reach the transitional region at 6°. Regarding the frictional region, when 1000 N is applied, the lateral force starts to decrease at 12°. However, when 750 N is applied, it starts to decrease at 11° approximately. The obtained results reveal that the vertical load directly affects the tire’s slip. 

**Figure 14:**
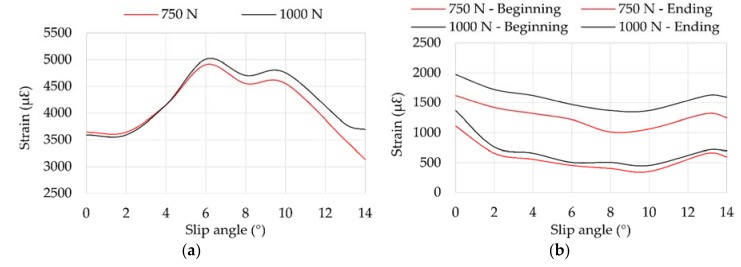
(**a**) Maximum strain peaks in channel 2 (ε_x_) at 0.8 bar, 30 km/h; (**b**) Maximum strain peaks in channel 3 (ε_y1_) at 0.8 bar, 30 km/h.

Figure 14 shows that the maximum strain data changes notably from 10°. In the case of channel 2, the maximum strain values practically halve in relation to channel 3. 

**Figure 15:**
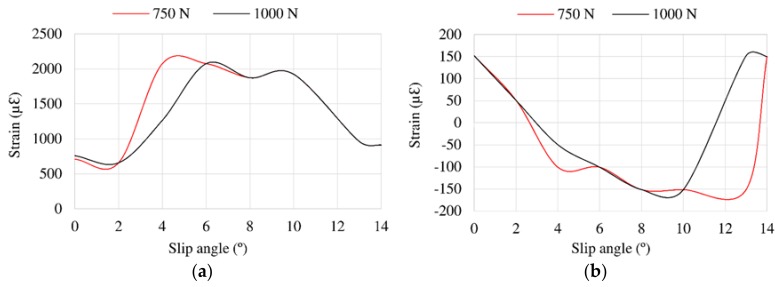
(**a**) Offset values in channel 2 (ε_x_); (**b**) Offset values in channel 3 (ε_y1_) for different vertical loads at 0.8 bar, 30 km/h.

Figure 15 illustrates the variation of offset values related to vertical load changes. In case of Figure 15b, the trends of strain curves change drastically from 10° for 750 N and from 12° for 1000 N. However, in longitudinal direction the trend’s change occurs from 10° in both cases.

## 4. Practical Approach to Estimate the Required Lateral Friction Coefficient.

In this section, a practical approach to show the potential of tire strain data to estimate the requested lateral friction coefficient (LFC) by the tire and detect the loss of grip is developed. Previous works have demonstrated that some key parameters of tire performance such as vertical load and slip angle can be accurately estimated by means of fuzzy logic. 

In this work, these estimated parameters are used to develop an estimation system from some input parameters, demonstrating that the tire’s loss of adhesion can be detected before reaching a critical situation for the driver and the required LFC by the tire can also be accurately estimated.

First of all, experimental *F_y_* data shown in Figure 13 is used to define an experimental model to calculate *F_y_* as a function of vertical load and slip angle by a single equation. Then, the fuzzy logic estimation system developed previously [38] and the experimental model are implemented in the environment MATLAB/Simulink^®^ (The Math-Works, Natick, MA, USA,) providing a signal to inform the driver and active control systems of the tire’s loss of grip. Finally, the requested LFC from fuzzy logic estimations and the estimated calculation of *F_y_* from the experimental model are used to calculate the required LFC from a theoretical equation.

### 4.1. Experimental Modelling of Lateral Force Behaviour

As is widely known, the lateral force depends mainly on vertical load and slip angle [14] , which have been accurately estimated from strain data in previous work [38], so the need now is to estimate the lateral force at the point where the tire loses grip. For this reason, the development of an experimental model to characterize and calculate the behavior of lateral force from vertical load and slip angle is especially interesting and it is essential to use the estimated values of those parameters in a real-time control system in the near future. In this section, the obtained *F_y_* curves (see Figure 13) for 750 N and 1000 N have been used to develop an experimental model of *F_y_* for the tire used in the tests. This equation, which defines the behavior of the lateral force through the linear and transitional regions (i.e., the range in which the tire is working at the limit of adhesion), is written as a function of *F_z_* and *α* as follows:

(1) $$F_{y}=C_{1}+\left(C_{2}\cdot F_{z}\right)\cdot \left(1-e^{\left(C_{3}\cdot \alpha \right)}\right)$$

where $C_{1}=10$, $C_{2}=0.889$ and $C_{3}=-1.06$.

This equation has been obtained by taking an exponential function as reference and then using regression techniques to adjust it to the *F_y_* experimental curves. This equation can be improved and adapted depending on the tested tire, but serves as a first approach to show the potential of the proposed methodology.

**Figure 16:**
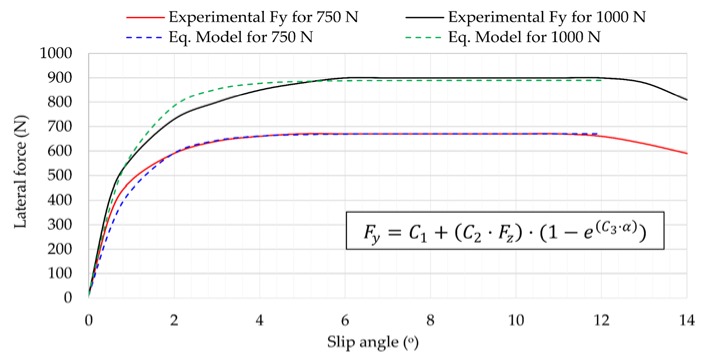
Experimental model adjusting to experimental *F_y_* data at 30 km/h, 0.8 bar.

Looking at the *F_y_* curves shown in Figure 16, they have an exponential shape that makes it possible to obtain an experimental model that fits quite well with the experimental data without considering too many parameters (the combined coefficient of correlation of the experimental model is 0.98). However, the behavior of *F_y_* for commercial tires can show curves with a shape more complicated to model mathematically.

Figure 16 also shows that the experimental equations do not fit with experimental data above 12°, where the tire starts sliding. Nevertheless, since the estimation system has been developed to detect the tire’s loss of grip before using this equation to obtain an estimated calculation of the required LFC, when the system detects the tire is sliding, it does not use the equation and returns a value of 0 for the LFC. In this way, the lack of fitting between the experimental model and the experimental data above 12° is not crucial. It should be highlighted that the 0 value of the LFC does not mean that the tire does not have good friction conditions to keep the current working conditions, but the tire is not working at the maximum lateral force hence the loss of control might be near if the conditions become more demanding, so the SDS could work as a warning system for drivers and active safety control systems to be ready to take actions. 

### 4.2. Simulink Implementation of Slip Detection System and Lateral Friction Coefficient Estimation

**Figure 17:**
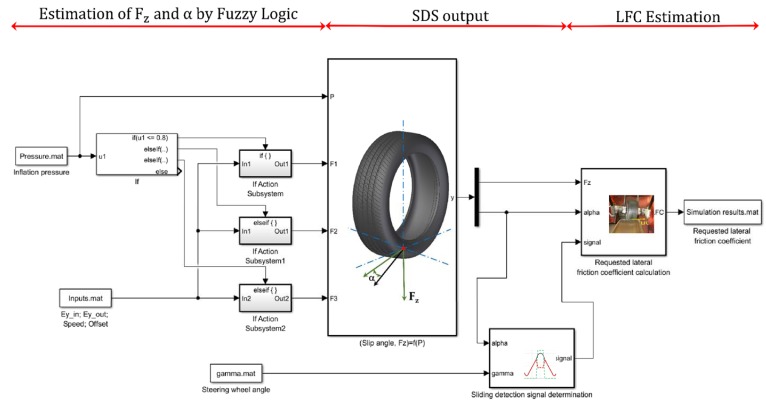
Slip Detection System implementation in MATLAB/Simulink^®^ and required lateral friction coefficient calculation.

The fuzzy logic system developed is implemented in MATLAB/Simulink^®^ to use the estimations of the vertical load and slip angle provided by the fuzzy logic system for detecting the tire’s slip by the SDS and estimating the required LFC, as shown Figure 17.

First, the fuzzy logic blocks developed are used to estimate vertical load and slip angle from several inputs. As a reminder, since the inflation pressure is an information accurately provided today by the Tire Pressure Monitoring System (TPMS) and rolling speed is also a known information, they are used as inputs. In addition, the fuzzy logic system uses the maximum strain peaks as well as the offset values (see Figure 7) as the main inputs to estimate vertical load and slip angle. 

Secondly, the wheel steer angle (gamma) is used as an input to calculate the tire steer angle, assuming that they could be monitored and they keep a relation of 1:15. Taking advantage of the fact that offset values for 13° and 14° give similar results as in case of 0°, 2° or 4° (see Figure 12a as an example), the fuzzy logic system estimates a value that does not correspond to the fixed wheel steer angle. In this way, as the estimations for the slip angle are quite accurate, it has been established that when the estimated slip angle differs by more than 10% of the tire steer angle, the SDS returns a value of 1 for the SDS output. As a first approach, it is assumed that the tire slip angle is directly related to the wheel steer angle, without considering the contribution of the other tires of the vehicle. This fact limits the application of the results taking into account the behavior of a whole vehicle, but this analysis serves as a proof of concept for the estimation of the vehicle slip angle from the corresponding equations of state. This simplification is not being considered in current developments. Finally, note that the relationship between lateral force and strain data is very advantageous, since strain data reduces drastically when a slight loss of grip occurs, therefore, the loss of grip can be detected before the situation becomes critical.

In the last stage, the estimated calculation of *F_y_* obtained by the experimental model defined in Equation (1) and the estimated value of *F_z_* obtained by fuzzy logic are used to calculate the required LFC by the following expression [41]:

(2) $$\mathrm{LFC}=\frac{F_{y}}{F_{z}}$$

Finally, it should not be forgotten that when the required LFC is calculated, the status of the tire’s sliding is known. Thus, when the SDS detects that the tire is losing grip (signal = 1), the Equation (2) is not used, and the value of the required LFC is automatically 0. 

### 4.3. Simulation Results

**Figure 18:**
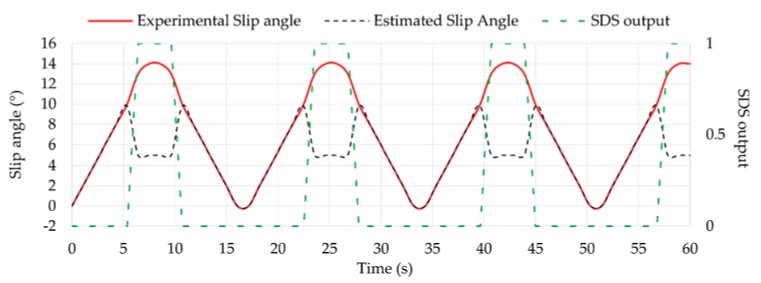
Tire slip detection from the slip angle estimation.

In this section, the results obtained in each stage of the implemented system in MATLAB/Simulink^®^ are illustrated. Figure 18 shows the slip angle estimated by fuzzy logic compared to the experimental slip angle, which is obtained using the relation that keeps with the wheel steer angle. As it is observed, when the tire is sliding, the estimated slip angle drops to 5° while the experimental slip angle has values of 13° and 14°. In these cases, the SDS output that indicates whether the tire is working or not is equal to 1.

**Figure 19:**
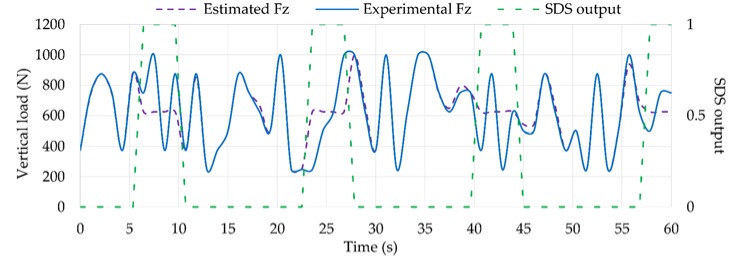
Simulation results of *F_z_* compared to experimental values.

During situations under slip working conditions (i.e., SDS output equal to 1), *F_z_* estimations by fuzzy logic compared to experimental *F_z_* data are not as accurate as when the tire is not sliding, as shown in Figure 19. 

**Figure 20:**
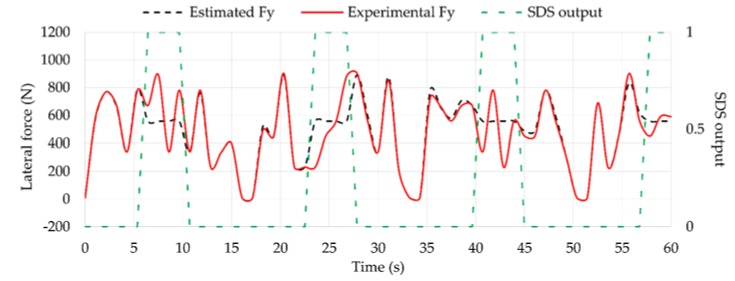
Simulation results of *F_y_* compared to experimental values.

In the same way, the estimated *F_y_* values using Equation (1) are worse in this situation, as shown in Figure 20. However, when the tire is working under optimum conditions (i.e., SDS output equal to 0), estimated *F_y_* and *F_z_* values are quite accurate compared to experimental data. Overall, the differences of estimated *F_z_* and *F_y_* compared with experimental data under optimum conditions are 1.64% and 3.11%, respectively.

**Figure 21:**
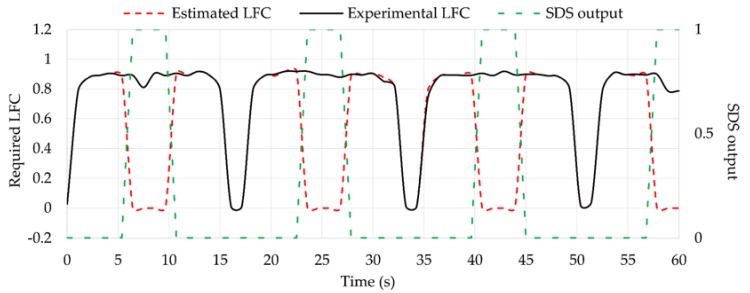
Estimated LFC compared to theoretical values.

Results obtained for the required LFC using the Equation (2) are shown in Figure 21. As they have been calculated from estimated *F_y_* and *F_z_* values, it is not considered as a direct calculation. However, results show that experimental LFC (obtained from experimental *F_z_* and *F_y_* data) and estimated LFC practically overlapped when the tire is working under optimum conditions (i.e., SDS output equal to 1), the percentage difference being around 1.5%. Finally, as mentioned before, the system implemented in Simulink returns a value of 0 for the required LFC when it detects the tire is losing grip, so the percentage differences between experimental and estimated values increase notably.

Finally, although it might be thought that the good slip angle estimations obtained (see Figure 18) when the tire is working within the limit of adhesion are not very useful, the sideslip angle of a vehicle, which is the ratio of forward and lateral velocities in the form of an angle, depends to a large extent on the tires’ slip angle [42]. The sideslip angle is essential for active safety control systems and its estimation from the tires’ slip angle is a work that needs to be further developed.

## 5. Discussion

In this work, tests in cornering rolling conditions at steady state (without evaluating demanding situations under acceleration or braking processes) have been carried out. The main equipment used was: data acquisition system, indoor test rig and a tire with attached strain sensors. The monitored information has been related not only with tire parameters such as vertical load, speed, pressure and slip angle, but also with the generated lateral force.

The obtained results reveal that tire working conditions affect, notably, the tire’s deformation. The variations of the considered test parameters imply not only changes in longitudinal and lateral maximum strain values, but also in the lateral force when slip angle increases. In addition, the tire’s loss of grip is shown in maximum tensile strain values, to such an extent that when the tire begins sliding, maximum strain value decreases or increases radically depending on the measuring direction. 

The intelligent tire used in this study has demonstrated that strain sensors are suitable for measuring tire dynamic behavior, particularly maximum tensile and compressive strain limits. Furthermore, the accuracy as well as the robustness of the strain gauges are demonstrated. Based on the results, strain gauges have demonstrated that they can measure with high accuracy the tire tread behavior under dynamic conditions.

The size of the strain gauges and the manufacturing processes of commercial tires gives a certain possibility to integrate the sensors into the tire layers, improving the robustness of the system and introducing the suggested strain-based methodology into mass production.

The possibility of detecting that the tire is working at the limit of adhesion before an accident occurs has been demonstrated by means of the implementation of the fuzzy logic systems in MATLAB/Simulink^®^. In addition, the experimental model to estimate the lateral friction coefficient has shown a good reliability compared to the theoretical calculation when the tire is working at the limit of grip. However, regarding the computational time, the developed estimation method must be optimized in order to meet the requirements of the real-time data acquisition. In addition, one of the limitations of the proposed model is that it would only work properly at the first stage of the understeer, since the relationship between the wheel steer angle and the tire slip angle remains constant only during this stage. Therefore, the analysis of the variation between these angles from certain values is part of the future works, since this information would provide more accurate estimations when the tire is working at the limit of adhesion. Moreover, in the same manner that other researches based on tire test rig did, it has been assumed that the wheel slip angle and the tire slip angle (that appears in the tire-road contact) are equivalent and have the same behavior. However, in order to develop a reliable system using the proposed methodology, it will be necessary to assess if it is possible to consider this assumption as correct, especially when tests under real conditions are carried out.

On the other hand, it needs to be emphasized that the model defined in Equation (1) is only valid for the steel drum. Different models should be applied for different road/tire combinations, which means that this work serves just as a proof of concept, but a reliable way must be found to predict the LFC in real time (for instance, by means of road image recognition). 

Future work will be focused on extending the range of some test conditions and testing other kind of tires, especially commercial tires, whose behavior is very different in comparison with slick tires. Secondly, although the proposed model serves as a proof of concept, tests considering different road conditions (i.e., friction coefficients) and considering other parameters (for instance, the tire and road temperatures) are necessary to corroborate some of the conclusions of this work and get a complete set of data that could allow us to estimate directly the lateral force instead of calculating it from the estimations of vertical load and slip angle. In addition, strain sensors attached to the sidewall could give us some useful information about tire working conditions. Finally, test under combined longitudinal and lateral slip conditions are necessary to decide if the proposed algorithm is suitable or other estimation methods such as Kalman filtering are more adequate before carrying out tests under real conditions.
